# Child maltreatment, peer victimization, and social anxiety in adulthood: a cross-sectional study in a treatment-seeking sample

**DOI:** 10.1186/s12888-019-2400-4

**Published:** 2019-12-27

**Authors:** Antonia Brühl, Hanna Kley, Anja Grocholewski, Frank Neuner, Nina Heinrichs

**Affiliations:** 1 Department of Psychology, Clinical Psychology and Psychotherapy, University of Bremen, Grazer Strasse 2, 28359 Bremen, Germany; 2 Department of Psychology, Institute of Clinical Psychology, Psychotherapy and Assessment, Outpatient clinic, Technische Universität Braunschweig, Humboldtstr. 33, 38106 Braunschweig, Germany; 3 Department of Psychology, Clinical Psychology and Psychotherapy, Outpatient clinic, Bielefeld University, Morgenbreede 2-4, 33615 Bielefeld, Germany

**Keywords:** Social anxiety, Social phobia, CTQ, Trauma, Child maltreatment, Peer victimization

## Abstract

**Background:**

Childhood adversities, especially emotional abuse, emotional neglect, and peer victimization are considered to be crucial risk factors for social anxiety disorder (SAD). We investigated whether particular forms of retrospectively recalled childhood adversities are specifically associated with SAD in adulthood or whether we find similar links in other anxiety or depressive disorders.

**Methods:**

Prevalences of adversities assessed with the Childhood Trauma Questionnaire (CTQ) and a questionnaire of stressful social experiences (FBS) were determined in *N* = 1091 outpatients. Adversity severities among patients with SAD only (*n* = 25), specific phobia only (*n* = 18), and generalized anxiety disorder only (*n* = 19) were compared. Differences between patients with anxiety disorders only (*n* = 62) and depressive disorders only (*n* = 239) as well as between SAD with comorbid depressive disorders (*n* = 143) and SAD only were tested.

**Results:**

None of the adversity types were found to be specifically associated with SAD and severities did not differ among anxiety disorders but patients with depressive disorders reported more severe emotional abuse, physical abuse, and sexual abuse than patients with anxiety disorders. SAD patients with a comorbid depressive disorder also reported more severe adversities across all types compared to SAD only.

**Conclusion:**

Findings indicate that particular forms of recalled childhood adversities are not specifically associated with SAD in adulthood. Previously established links with SAD may be better explained by comorbid depressive symptoms.

## Background

Childhood maltreatment and peer victimization are known to be crucial risk factors for mental health [1]. In social anxiety disorder (SAD), social learning experiences in childhood and adolescence are an important component of contemporary etiological models [1–3]. Therefore, many researchers investigated the link between childhood maltreatment and SAD and repeatedly showed that exposure to such experiences in childhood are associated with SAD in adulthood [4, 5]. However, comparing results across studies is difficult because a variety of assessments have been employed to assess childhood maltreatment.

A growing body of research investigated the association between history of childhood maltreatment and SAD by using the Childhood Trauma Questionnaire (CTQ) [6], which assesses emotional abuse, physical abuse, sexual abuse, emotional neglect, and physical neglect. These studies linked specific maltreatment types to SAD symptom severity in individuals with SAD [4, 5, 7, 8] and/or compared histories of child maltreatment between individuals with SAD and non-clinical control groups [5, 9]. Findings showed that emotional abuse [5, 9] and emotional neglect [5] were more often reported by subjects suffering from SAD in comparison with healthy controls. Moreover, emotional abuse and emotional neglect have been significantly associated with greater SAD symptom severity [4, 5, 7, 8], even in non-clinical samples [10, 11]. One study [5] further found that emotional abuse and neglect were linked to greater severity of depressive symptoms. Fewer studies found significant associations of physical neglect [4] and sexual abuse [7, 9] with SAD.

In addition to childhood maltreatment types, where perpetrators usually include parents and other adults, peer victimization is considered to be a further adverse social learning experience contributing to the development of SAD. In the interactional model of SAD, Spence and Rapee propose that exposure to peer victimization may increase the risk of developing SAD in individuals “who are intrinsically vulnerable “ ([1], p., 8) (e.g., due to a genetic predisposition and/or inhibited temperament), through their impact upon behavioral and cognitive factors, including avoidance behavior or maladaptive schemes.

Therefore, a child exposed to peer victimization is more likely to experience social interactions as harmful, which may reinforce negative beliefs about themself and relationships with peers. This may lead to the avoidance of social interactions, and thereby increasing levels of social anxiety [1, 12]. Indeed, studies have shown that peer victimization, including threats or acts of physical aggression (*overt victimization*), relational manipulation or social exclusion (*relational victimization*), and damaging a peers’ reputation (*reputational victimization*) is associated with social anxiety symptoms in adolescence and adulthood [13–15]. Growing evidence from prospective investigations implies that peer victimization puts children and adolescents at risk for developing social anxiety [12, 14, 16, 17], with findings differing across types of victimization. Especially relational victimization, which is typically initiated by friends [18] and comprises behavior such as excluding someone or withholding a relationship, is most strongly associated with social anxiety compared to overt or reputational victimization [14]. However, cross-sectional studies further suggest that socially anxious youth are also more likely to become targets for peer victimization [17, 19–21], so that peer victimization seems to constitute both, a predictor as well as a consequence of social anxiety [12, 14, 17, 22].

Preliminary studies, which investigated peer victimization and childhood maltreatment simultaneously, report inconsistent findings. While cross-sectional data [23] implies that emotional child maltreatment and peer victimization are independently linked to SAD symptom severity, longitudinal data [24] suggest that emotional peer victimization, but not parental emotional abuse, increases social anxiety symptoms. In this longitudinal study [24], emotional peer victimization was assessed with the items on relational victimization from the victimization scale of the Peer Reactions Questionnaire [25].

In sum, the balance of evidence to date demonstrates that particular forms of recalled childhood adversities, namely emotional abuse, emotional neglect, and peer victimization may have more impact on SAD than other forms of childhood adversities. However, one of the key questions concerning effects of childhood adversities on SAD is the specificity of these effects. In other words, does having experienced emotional abuse, emotional neglect, or peer victimization increase the risk for SAD specifically, any anxiety disorder, any affective disorder, or any psychopathology? The above summarized studies on child maltreatment usually recruited participants for specific research projects, e.g. a brain imaging studies [5] or intervention trials [4, 7, 8], in which high internal validity is requested. Comorbidity was therefore allowed only on a limited basis. In some studies, depressive disorders were allowed [4, 8, 9], in others, these were excluded [5, 7]. Assessing comorbidity, however, may be highly relevant for the conclusions of such investigations.

For example, Rapee [26] reviewed the evidence for specificity of sexual or physical abuse as a risk factor for anxiety disorders and concluded that “sexual abuse is shown to be a risk factor for a variety of forms of psychopathology “(p. 73). In fact, some of the effects of sexual abuse may rather unfold in patients with comorbidity of anxiety and affective disorders rather than in “pure” anxiety disorders only (p. 40) [27]. Beyond that, exposure to childhood adversities is associated with a range of mental health problems in later life. For example, child maltreatment has shown to be associated with mood and anxiety disorders, substance abuse, psychotic symptoms, and personality disorders [28]. Similarly, peer victimization does increase the risk for several dimensions of psychopathology, specifically internalizing problems [29–31]. Only preliminary evidence suggests that social anxiety and not depression may be specifically linked to peer victimization [13].

Taken together, key limitations in previous studies include a) the unclear specificity of the effects of childhood adversities on SAD. Most previous studies investigated associations with SAD symptom severity in individuals with SAD but did not examine whether these links are specific to a SAD diagnosis compared to other disorders. b) The neglected role of comorbid disorders in these effects, and c) limited generalizability of previous results to clinical treatment-seeking samples with high external validity (individuals not participating in a randomized controlled trial or recruited for a specific research project). Given the evidence that patients exposed to childhood adversities show poorer treatment outcomes [32], research in representative treatment-seeking samples is key in informing practitioners and developing treatment interventions for patients commonly seen in out-patient clinics.

Therefore, the primary aim of the present study is to examine whether particular forms of recalled childhood adversities, namely emotional abuse, emotional neglect, and peer victimization are specifically associated with SAD in adulthood or whether we find similar links in other anxiety or depressive disorders by using a clinical sample who is seeking psychotherapy more in a routine care setting instead of a randomized controlled trial.

In order to investigate the specificity of links between recalled childhood adversities and SAD, we hypothesize that (1) recalled childhood emotional abuse, emotional neglect, and peer victimization are more likely to be associated with SAD than with any other mental disorder while controlling for comorbidity, (2) childhood adversity severities will differ across SAD, specific phobia (SP), and generalized anxiety disorder (GAD) without comorbidities, and (3) childhood adversity severities will differ between anxiety disorders and depressive disorders without comorbidities. The secondary aim of this study is to clarify the role of comorbid depressive disorders in the assumed effects of recalled childhood adversities. Therefore, (4) we expect that patients with SAD and comorbid depressive disorder will report more childhood adversities than patients with SAD only.

## Methods

### Participants

Data for the present cross-sectional study were obtained from *N* = 1091 treatment-seeking outpatients, who completed the CTQ before the initiation of psychotherapy treatment. All patients were assessed in a routine care setting in one of two German outpatient clinics affiliated with the University of Braunschweig (*n* = 218) or Bielefeld University (*n* = 873). Eligible patients were 1) at least 18 years old, 2) met the criteria for at least one mental health diagnosis according to the Diagnostic and Statistical Manual of Mental Disorders (DSM-IV TR) [33], and 3) provided their data for research purposes. Primary diagnoses of all patients (690 women, 401 men, *M*_age_ = 34.83, *SD* = 12.11) included 42% depressive disorders and 21% anxiety disorders (within 32% with SAD). Further diagnoses included reaction to severe stress, and adjustment disorders (13%), eating disorders (5%), obsessive-compulsive disorder (4%)**,** personality disorders (4%), somatoform disorders (4%), disorders due to psychoactive substance (1%), schizophrenia, schizotypal and delusional disorders **(**1%)**,** and other disorders (6%; e.g., sleep disorders, sexual dysfunction**,** habit and impulse disorders**,** dissociative conversion disorders, and hyperkinetic disorders)**. A**bout half of the sample (52%) had at least one comorbid disorder. The majority of the sample (44%) had a part-time or full-time job, while 31% were students or apprentices, 3% were housewives, 7% were retired or unemployable, and 11% were unemployed (4% did not respond to this question). Most patients (84%) were presently married or in a relationship, 5% were single, and 11% were divorced or separated.

For the primary aim of our study, we examined different subgroups of patients with: (a) SAD with or without any comorbidity (SAD+/−, *n* = 171) and patients with any other mental disorder with or without comorbidity (OD+/−, *n* = 801). We further compared groups that still indicated acceptable cell numbers for analyses after excluding patients with comorbidities: (b) patients with SAD only (SAD-, *n* = 25), GAD only (GAD-, *n* = 19), SP only (SP-, *n* = 18), and (c) patients with an anxiety disorder only (AD-, *n* = 62; i.e. SAD-, SP- or GAD-) versus patients with a depressive disorder only (DEP-, *n* = 239; i.e. depressive episode, recurrent depressive disorder, or dysthymia). For the secondary aim of the study, we compared patients with SAD and a comorbid depressive disorder (SAD+DEP, *n* = 143) versus SAD only (SAD-, *n* = 25).

### Measures

#### Assessment of diagnosis

Diagnostic data were obtained using the German version of the Structured Clinical Interview for DSM-IV (SCID I) [34] to diagnose major mental disorders (DSM-IV Axis I) according to the Diagnostic and statistical manual of mental disorders [33]. The SCID for Axis I is routinely used in both clinics. All interviewers were trained and either licensed therapists or those in training undergoing supervision.

#### Assessment of child maltreatment

Retrospective data on recalled child maltreatment were assessed with the German version of the Childhood Trauma Questionnaire (CTQ) [6, 35]. The 28-item version of the CTQ contains five subscales to assess different forms of trauma: emotional abuse, physical abuse, sexual abuse, emotional neglect, and physical neglect. Each of these subscales consists of 5 items, such as “people in my family said hurtful or insulting things to me” (emotional abuse). Each item is rated from 1 (*never true*) to 5 (*very often true*), so that subscale scores can range from 5 to 25 with higher scores indicating more severe maltreatment. The continuous subscale scores were used to test our hypotheses. The employed version further contained 3 items to assess minimization/denial indicating a positive response bias. Given that the subscale physical neglect shows insufficient internal consistence and high intercorrelations with the other subscales [36], only the first four subscales were used to test our hypotheses. In order to describe frequency rates of maltreatment forms, cut-offs for each subscale were applied according to Walker and colleagues [37]: emotional abuse ≥10, physical abuse ≥8, sexual abuse ≥8, and emotional neglect ≥15. However, frequency rates were not used to test our hypotheses due to reduced cell sizes. The German version has been shown to be a reliable and valid measure to screen for these four forms of childhood maltreatment. Wingenfeld and colleagues [35] found good psychometric properties, including high internal consistency of all trauma scales with Cronbachs α ≥ 0.89, except for physical neglect with α = 0.62. Similar, internal consistencies in our study were high to excellent, with Cronbach’s α = .88 (emotional abuse), α = .86 (physical abuse), α = .96 (sexual abuse), α = .91 (emotional neglect), except for physical neglect (α = .61).

#### Assessment of peer victimization

The Questionnaire of Aversive Social Experiences in the Peer Group (Fragebogen zu belastenden Sozialerfahrungen in der Peer Group, FBS) [38] was employed to retrospectively assess the exposure to various forms of peer victimization. On a list of 22 aversive social situations, such as being excluded, insulted or laughed at (e.g., “It happened that everyone was invited to a party, but not me”), patients reported whether they had experienced this situation or not (*Yes* or *No*) during childhood (age 6–12) or adolescence (age 13–18). In this study, the total sum score of all *Yes* responses (0–44) was used for analyses. Preliminary evaluations report satisfying psychometric properties, with solid stability over a period of 20 months and good construct validity [38]. Moreover, findings suggest that the FBS may assess a distinct form of child maltreatment, due to its incremental contribution to the prediction of psychopathology beyond child maltreatment assessed with the CTQ [31]. In our study, we obtained excellent internal consistencies for the FBS childhood scale (Cronbach’s α = .90), the adolescence scale (α = .96), and the total sum score (α = .97).

### Procedure

The study was approved by the ethics committee of the University of Braunschweig. Data for this cross-sectional investigation was collected in two outpatient clinics within the scope of the routine diagnostic assessment between 2013 and 2018. The standard routine assessment comprised structured clinical interviews and a battery of self-report questionnaires, assessing participants’ demographics, mental health, and psychosocial functioning. For research purposes only, patients further completed the CTQ and the FBS in the outpatient clinic of the University of Braunschweig. In the outpatient clinic of Bielefeld University, the CTQ was already included in the routine diagnostic assessment and only the FBS was completed for research purposes only. Self-report questionnaires were completed via a paper-pencil version. Patients provided their written consent for using the anonymized data for research. A list of all available measures employed in this research project can be obtained from the second and third authors.

### Data analyses

Analyses included the entire sample for whom CTQ and/or FBS responses were available (*N* = 1091). Missing data analyses indicated a systematically missing of values so that we did not impute missing values in our study (see Additional file 1A). In cases where the assumptions for parametric analyses were violated, non-parametric tests were employed. Demographic characteristics, rates of child maltreatment, and peer victimization are descriptively reported with means (*M*) and standard deviations (*SD*) for continuous variables as well as counts for categorical variables. Demographic group differences were tested with *χ*^2^ analyses and independent t-tests. Further preliminary analyses included the calculation of the CTQ minimization/denial severity across patient groups (see Additional file 1B). Intercorrelations among demographic characteristics, child maltreatment scales, and peer victimization were calculated using Spearman r_s_ (see Additional file 1C).

In order to investigate, whether particular forms of recalled childhood adversities are more likely to be associated with SAD than with any other mental disorder, we examined the odds of developing SAD+/− by conducting a binary logistic regression with age, gender, occurrence of comorbidity, CTQ maltreatment scales, and peer victimization as independent variables (forced entry method). All CTQ subscales were included in the analysis of hypothesis 1 nevertheless we expected based on the literature that emotional abuse, emotional neglect, and peer victimization are more likely to be associated with SAD+/−. We controlled for participants´ age and gender by including these variables into the model because participant’s age significantly differed between our groups (see Table 1) and gender differences in social anxiety have been established [39]. Results of Nagelkerke’s *R*^2^ and Hosmer-Lemeshow test are reported to evaluate the goodness of fit of the model. In order to find an anticipated *R*^*2*^ = 0.10, OR 1.8 with a power of 95%, a sample size of *n* = 328 was required for the logistic regression. Given that the homogeneity of covariance matrices could not be assumed, multivariate tests could not be implemented. Instead, we used multiple tests for independent samples for examining whether effects of childhood adversities are specific to SAD. Kruskal-Wallis tests were employed to compare childhood adversities among the patient groups SAD-, SP-, and GAD-. Due to reduced statistical power, these results should be interpreted cautiously. Independent t-tests were computed to investigate differences in childhood adversities between AD- and DEP-. In order to compare the patient groups SAD- and SAD+DEP, further t-tests were employed. Effect sizes Hedge’s *g* were computed for each comparison by adjusting the calculation for different sample sizes. Given the multiple testing, alpha levels were Bonferroni adjusted for the number of tests (i.e. adjusted alpha: *p* = .0028). All analyses were carried out with SPSS 24.

**Table 1:** Demographic Characteristics, Child Maltreatment and Peer Victimization in the total Sample, Patients with SAD, and Patients with other Disorders

Characteristics	Total (*n* = 1091)		SAD+/− (*n* = 192)		OD+/− (*n* = 899)		
	*n*	(%)	*n*	(%)	*n*	(%)	*χ*^2^Test (*p*)
Gender							.371
Male	401	36.76	76	39.58	325	36.15	
Female	690	63.24	116	60.42	574	63.85	
Child Maltreatment							
Emotional abuse	497	46.80	87	47.03	410	46.75	.945
Physical abuse	273	25.30	51	26.84	222	24.97	.590
Sexual abuse	179	16.70	27	14.21	152	17.23	.311
Emotional neglect	422	39.89	77	41.62	345	39.52	.596
	*M*	*SD*	*M*	*SD*	*M*	*SD*	*t*-Test (*p*)
Age	34.83	12.11	31.29	10.00	35.60	12.39	< .001
Peer Victimization	11.85	8.38	13.53	8.73	11.49	8.26	.003

*Note. M* Mean, *SD* Standard Deviation, *SAD+/−* Social anxiety disorder with or without comorbidity, *OD+/−* Other disorders with or without comorbidity. Child maltreatment threshold for clinical significance in CTQ established by Walker et al. [37]

## Results

### Preliminary analyses

Table 1 shows demographic characteristics, child maltreatment rates, and peer victimization for the total sample (*N* = 1091), patients with SAD (87% with comorbidities) and patients without SAD. Descriptive analyses showed emotional abuse as the most commonly reported type of child maltreatment (46%), followed by emotional neglect (39%), physical abuse (25%), and sexual abuse (16%).

Table 2 summarizes the extent and severity of childhood maltreatment in patients with SAD only as well as patients with SAD and comorbidities in our study. Severities and rates found in other clinical studies, including a large sample of German in- and outpatients [35] as well as a representative sample drawn from the general German Population [40], can be found in the Additional file 1D.

**Table 2:** Frequencies and Severities of Child maltreatment assessed with the CTQ in Social Anxiety Disorder

		Emotional Abuse		Physical Abuse		Sexual Abuse		Emotional Neglect	
	*N*	*M (SD)*	*%*	*M (SD)*	*%*	*M (SD)*	*%*	*M (SD)*	*%*
SAD-	25	8.12 (2.88)	28.00	5.24 (0.72)	4.00	5.04 (0.20)	0.00	11.14 (3.95)	28.00
SAD+/−	192	10.92 (5.43)	47.03	7.06 (3.68)	26.84	6.49 (4.11)	14.21	13.60 (5.88)	41.62

*Note*. *M* Mean, *SD* Standard Deviation, *%* Percentage of participants meeting the threshold for clinical significance, *CTQ* childhood trauma questionnaire. Threshold for clinical significance in CTQ established by Walker et al. [37]. SAD- = Social anxiety disorder only, SAD+/− = Social anxiety disorder with or without comorbidity

### Emotional abuse, emotional neglect, and peer victimization are more likely to be associated with SAD than with other mental disorders (hypothesis 1)

The results of the binary logistic regression are presented in Table 3. A total of *n* = 972 cases were analyzed and the full model significantly predicted SAD+/− (omnibus χ^2^ = 137.94, *df* = 8, *p* < .001; Hosmer-Lemeshow Test χ^2^ = 2.72, *df* = 8, *p* = .950). Nagelkerke’s *R*^*2*^ indicated that the model accounted for 22% of the variance in SAD+/−. Age and the presence of comorbidity predicted SAD+/−. The values of the coefficients revealed that an increase of 1 year in age is associated with a decrease in the odds of SAD+/− by a factor of 0.97 (95% CI 0.95 and 0.99). For patients without comorbidities, the chance of SAD+/− decreases by a factor of 0.12 (95% CI 0.07 and 0.19) when compared to patients with comorbidities. Neither emotional abuse, emotional neglect nor peer victimization was associated with SAD.

**Table 3:** Results of the Logistic Regression for SAD Diagnosis

Independent variable	*p* value	Odds-Ratio	95% CI
Age	<.001	.97	[0.95, 0.99]
Gender	.062	1.44	[0.98, 2.11]
Comorbidity	<.001	.12	[0.07, 0.19]
Emotional abuse	.767	1.01	[0.96, 1.06]
Physical abuse	.127	.95	[0.88, 1.02]
Sexual abuse	.289	.97	[0.93, 1.02]
Emotional neglect	.586	1.01	[0.97, 1.05]
Peer victimization	.348	1.01	[0.99, 1.04]

Note. *N* = 972 patients (*n* = 171 SAD+/−, *n* = 801 = OD+/−) were included in the analysis. Criterion: diagnosis (0 = other disorders OD+/−, 1 = SAD+/−). Independent variables: Age in years, gender (0 = male; 1 = female), comorbidity (0 = comorbidity, 1 = no comorbidity), emotional abuse (CTQ scale), physical abuse (CTQ scale), sexual abuse (CTQ scale), emotional neglect (CTQ scale), peer victimization (FBS total score). CI = confidence interval

### Childhood adversity severities will differ across SAD, SP, and GAD only (hypothesis 2)

**Fig. 1:**
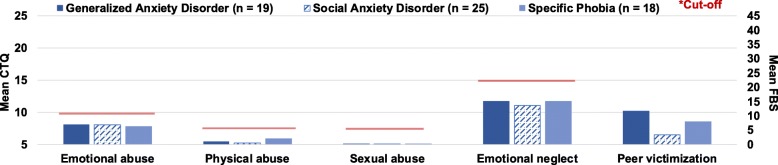
Group differences in means of childhood adversity severity across SAD-, SP-, and GAD-. Emotional abuse, physical abuse, sexual abuse, and emotional neglect assessed with the CTQ; Peer victimization assessed with the FBS; Cut-off according to Walker et al. [37]. Means and standard deviations can be found in the Additional file 1E1

The Kruskal-Wallis tests revealed that the patient groups SAD-, SP-, and GAD- did not differ in their severity of emotional abuse (χ^2^ [2, *N* = 62] = 0.60, *p* = .741), physical abuse (χ^2^ [2, *N* = 62] = 2.61, *p* = .272), sexual abuse (χ^2^ [2, *N* = 62] = 0.91, *p* = .634), emotional neglect (χ^2^ [2, *N* = 62] = 0.15, *p* = .930), or peer victimization (χ^2^ [2, *N* = 60] = 2.29, *p* = .318) with the adjusted alpha *p* = .0028. Figure 1 illustrates these findings.

### Childhood adversity severities will differ between anxiety and depressive disorders (hypothesis 3)

**Fig. 2:**
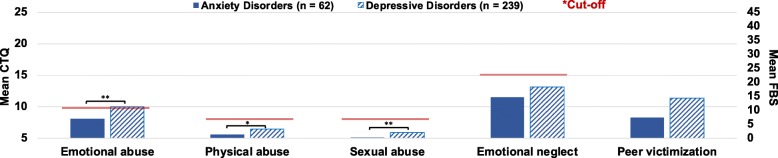
Group differences in means of childhood adversity severity between anxiety disorders and depressive disorders without comorbidity. Emotional abuse, physical abuse, sexual abuse, and emotional neglect assessed with the CTQ; Peer victimization assessed with the FBS; Cut-off according to Walker et al. [37]. Means and standard deviations can be found in the Additional file 1E2. * *p* < .01 ** *p* < .001

Multiple independent t-tests showed that the DEP- group reported significantly more severe emotional abuse (t = − 3.64, *df* = 137.63, *p* < .001, g = .42), physical abuse (t = − 2.91, *df* = 149.79, *p* = .004, g = .32), and sexual abuse (t = − 4.51, *df* = 286.27, *p* < .001, g = .35) compared to patients with AD- (adjusted alpha: *p* = .0028). No significant differences emerged for emotional neglect (t = − 2.07, *df* = 297, *p* = .039, g = .30) and peer victimization (t = − 2.83, *df* = 285, *p* = .005, g = .41, see Fig. 2).

### Patients with SAD and comorbid depressive disorder will report more childhood adversity severities than patients with SAD only (hypothesis 4)

**Fig. 3:**
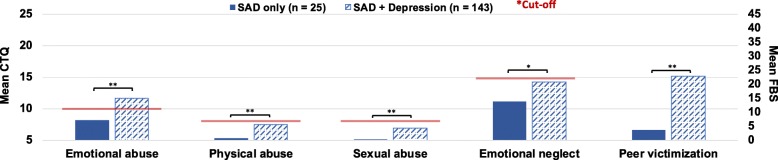
Group differences in means of childhood adversity severity between SAD only and SAD with comorbid depressive disorder. Emotional abuse, physical abuse, sexual abuse, and emotional neglect assessed with the CTQ. Peer victimization assessed with the FBS; Cut-off according to Walker et al. [37]. Means and standard deviation can be found in the Additional file 1E3. * *p* < .01 ** *p* < .001

Results of further independent t-tests showed that patients with SAD+DEP reported significantly more severe emotional abuse (t = − 4.65, *df* = 65.09, *p* < .001, g = 0.65), physical abuse (t = − 6.00, *df* = 165.83, *p* < .001, g = 0.59), sexual abuse (t = − 4.81, *df* = 144.93, *p* < .001, g = 0.44), emotional neglect (t = − 3.23, *df* = 46.38, *p* = .002, g = 0.53), and peer victimizations (t = − 6.68, *df* = 47.55, *p* < .001, g = 1.01) than patients with SAD- (Fig. 3).

## Discussion

The primary aim of the study was to investigate whether effects of different forms of recalled child maltreatment and peer victimization on SAD are specific to SAD or whether we find similar effects in other disorders as well. Four key findings emerged. Contrary to our expectations, none of the different child maltreatment types or peer victimization were found to be predictive for a SAD diagnosis in adulthood in an exclusively clinical sample. Thus, none of these childhood adversities seem to be more likely associated with SAD than with other disorders in the present sample. These findings seem inconsistent with previous findings, which may be partially explained by differences in the study design. Previous studies usually investigated the associations between childhood adversities and symptom severity in SAD samples [4, 8] and/or compared SAD patients with healthy controls [5], whereas we investigated links between childhood adversities and a categorical diagnosis, assessed with a clinical interview.

Secondly, neither any form of child maltreatment nor peer victimizations significantly differed among patients with SAD, SP, or GAD without comorbidities. Although previous studies repeatedly showed that at least childhood emotional abuse and emotional neglect seems to be strongly linked to SAD severity in adulthood [4, 5, 7–9], these effects may not be specific to SAD, but rather apply for other anxiety disorders as well. Indeed, preliminary research indicates that child maltreatment as well as peer victimization are associated with an increased risk for any anxiety disorder, including SP and GAD [15, 30, 41–43]. However, studies investigating links between other anxiety disorders than SAD or PTSD and child maltreatment assessed with the CTQ are scarce, limiting the comparability with our results.

The third key finding implies that effects may not only be non-specific to SAD, but broader non-specific to anxiety disorders. Comparing patients with anxiety disorders and depressive disorders without comorbidities showed that patients did not differ in severities of recalled emotional neglect and peer victimization. Patients with a depressive disorder reported significantly more severe emotional abuse, physical abuse, and sexual abuse. Our results support previous findings that child maltreatment constitute a risk factor for both, any anxiety disorder and any depressive disorder, although somewhat stronger association emerge for child maltreatment with depressive disorders than with anxiety disorders [26, 44, 45]. Moreover, a recent study [46] investigated which forms of child adversities are the best predictors for the development of lifetime major depressive disorder in women with or without depression (thus with other disorders or healthy ones). Especially emotional neglect and parental non-verbal emotional abuse assessed with the German interview version of the MACE (KERF-I) [47] were the best predictors of lifelong depression. Taken together, our results are in line with the balance of evidence to date, implicating that consequences of particular maltreatment forms, i.e. the emotional forms and peer victimization are not specific to SAD, but expand to a wide range of other mental disorders, including internalizing problems, depression, risky health behavior, or eating disorders [16, 26, 30, 48, 49].

In line with our hypotheses, the fourth key finding of our study showed that patients with SAD and a comorbid depressive disorder reported significantly more severe child maltreatment and peer victimization on all scales than patients with SAD only. Considering that several studies did not sufficiently control for comorbidities, this finding may contribute to the explanation of inconsistencies in the literature on effects of childhood adversities on SAD. We propose that the effects of emotional adversities on SAD stated in the literature are not specific to the disorder and moreover, may be better explained by the even stronger associations between childhood adversities and depressive disorders. Given the stated group differences in patients with anxiety and depressive disorders, these findings do not seem surprising. Indeed, emotional neglect has been shown to be associated with higher depression severity and lower self-esteem in patients with SAD [5]. Moreover, preliminary findings showed that the association between sexual abuse and anxiety disorders only emerged in patients with a comorbid depressive disorder [27]. Results on peer victimization are further in line with findings from Ranta and colleagues [13], who found that among boys, social anxiety with comorbid depressive symptoms were more strongly linked to all forms of peer victimization than depression or social anxiety alone. Among girls, only the relational victimization was more frequent in the comorbid group than in the social anxiety or depression only groups. Other findings suggest that a relationship between peer victimization and anxiety is not attributable to a diagnostic overlap between anxiety and depression [15]. However, measures to assess peer victimization vary immensely across studies and as yet there is insufficient evidence to draw valid implications on how peer victimization may contribute to the development of SAD beyond transdiagnostic effects.

Given the high comorbidities between SAD and depressive disorders, more research is warranted to investigate whether SAD is a cause or effect of comorbid disorders, or as previously discussed, “whether these patterns of comorbidity reflect common underlying causal factors” (p. 51) [1]. Epkins and Heckler [50] described that several models of both disorders in youth incorporate family-related problems as well as dysfunctional relationships with peers or adults, including social isolation, rejection, or criticism, associated with low self-esteem, social withdrawal, loneliness, and difficulties in interpersonal problem-solving. Therefore, both SAD and depressive disorders comprise interpersonal processes in the development as well as interpersonal consequences, indicating overlapping constructs in models of both disorders. Initial theories [51] expected that adverse emotional events related to loss were more specific for depression, while direct threatening events such as physical or sexual abuse were more related to anxiety [52]. However, we suppose that specific pathways from childhood adversities to SAD or depression may not be specific to the disorder, but rather lead to specific symptoms in specifically predisposed individuals. For example, in the frame of schema-based cognitive model of depression and anxiety [53], it has been shown that emotional neglect was strongly associated with two out of three symptom dimensions (general distress and anhedonic depression), whereas sexual abuse was associated with also general distress and anxious arousal independent of an anxiety or depressive diagnosis [54]. Further nonclinical findings suggest that emotional adversities are more strongly linked to internally-focused symptoms, while sexual and physical abuse are more associated with externally focused symptoms [55].

Teicher and Samson [56] point at the difference between survivors of early maltreatment and other individuals with the same mental disorder: they characterize disorders in maltreated individuals to be of greater severity, with more comorbidity, and less favorable treatment response. In fact, these authors are suggesting a “critically distinct subtype across depressive, anxiety, and substance use disorders” (p. 1114) for individuals with childhood maltreatment, defined as an “ecophenotype”. Therefore, an interesting approach for future research may be to investigate the effects of childhood adversities on a symptom level rather than a diagnostic level. A network perspective of psychopathology [57], by conceptualizing disorders as casual networks of mutually reinforcing symptoms, may be a promising approach to investigate how childhood adversities and their potentially intercorrelated pathways affect transdiagnostic symptoms, to identify clusters of potential ecophenotypes, and to shed light on how these risk factors may contribute to the development of SAD and other disorders.

### Strengths and limitations

To our knowledge this is the first study that made efforts to compare the recalled childhood adversities for patients diagnosed with anxiety and depression with or without comorbidities according to the gold standard in routine clinical care. In contrast to most previous studies, generalizability of our results to treatment-seeking samples can be assumed. Our investigations in routine clinical care provide valuable and representative information on prevalence rates and impacts of childhood adversities for treatment-seeking patients in Germany. Therefore, this information can guide practitioners in understanding the role of childhood adversities in the development of mental disorders.

Given that our sample was not recruited for a specific treatment study, anxiety patients without comorbidities were rare in this sample. However, this is truly characteristic of the out-patient population in Germany. Although comparing “pure” diagnostic groups may be considered a strength of our study, the cell number reductions due to anxiety patients with comorbidities were detrimental, limiting our statistical validity. Therefore, the null results may be attributable to low statistical power arising from small sample sizes. The small sample size of SAD only patients (*n* = 25) may further challenge if this group is truly representative of this subpopulation. While child maltreatment severities and rates in SAD patients with comorbidities were comparable to other SAD+/− samples [4, 7] and a depressive sample [35], SAD only patients reported even lower severities of physical abuse and sexual abuse than a representative sample of the German population [40]. However, treatment-seeking samples in routine care settings come with these kinds of data limitations. To our knowledge, maltreatment rates (assessed with the CTQ) in SAD only groups from other studies are lacking. Our result may encourage future research to fill this gap in the literature and further investigate effects in other anxiety disorders besides SAD and PTSD, with larger and pure diagnostic groups.

Although most previous studies investigated associations with SAD symptom severity in patients with SAD, we did not use symptom severity ratings in our analysis. Due to our representative treatment-seeking sample with different mental disorders, we did not assess SAD symptom severity in every patient. Our study further focused solely on the comorbidity with depressive disorders. It remains unclear, how other comorbidities may affect associations with childhood adversities. Preliminary studies showed that patients with SAD and comorbid ADHD report more emotional abuse and emotional neglect than patients with SAD only [58]. Meanwhile, no difference emerged in patients with SAD with and without schizophrenia [9]. Future studies may pay more attention to effects on transdiagnostic symptoms by systematically controlling for several comorbidities.

Finally, causal inferences about the links between childhood adversities and SAD are limited by the cross-sectional nature of our study. Thus, childhood adversities have been retrospectively assessed, which is prone for a recall bias and social desirability effects. Indeed, 22% of patients endorsed at least one minimization/denial item on the CTQ and our missing data analyses revealed that patients with missing values on the emotional abuse, physical abuse, and sexual abuse scales reported significant higher maltreatment severities on other maltreatment scales compared to patients without missing values. Therefore, maltreatment rates and severities reported in our sample are likely to be underestimated. Given that preliminary evidence indicates that individuals with depression show more negative bias in memory and attention compared to individuals with SAD [59], depressed patients in our study may have recalled more adverse childhood memories and thus, reported more severe maltreatment. The retrospective assessment further prevents implications on interplays among adversities across the life span. For instance, maltreatment by parents in childhood seems to be associated with a higher risk for later peer victimization [60, 61]. Longitudinal studies assessing the detailed temporal order of adversities are needed to identify potential mediating mechanisms across adversities to explain potential early pathways leading to SAD in later life.

## Conclusion

Although links between forms of childhood adversities and SAD in adulthood have been established, our findings indicate that these effects are not specific to SAD, but rather apply to other anxiety and depressive disorders as well. Moreover, our findings implicate that most recalled childhood adversities are stronger associated with depressive disorders than with anxiety disorders. Finally, we conclude that some effects of specific childhood adversity types on SAD stated in the literature may be better explained by comorbid clinical or non-clinical depressive symptoms or may be solely based on severity of anxiety symptoms than on disorder type.

Taken together our findings support that recalled childhood adversities, including emotional abuse, emotional neglect, and peer victimization constitute transdiagnostic risk factors for a range of mental disorders in adulthood. In order to explain this multifinality, future studies may investigate effects of particular forms of childhood adversities on transdiagnostic outcomes in individuals with specific vulnerabilities, instead of continuing to examine links with single disorders. Identifying potentially moderating individual differences and vulnerabilities, as well as mediating psychological and interpersonal mechanisms, would be of particular value in informing the development of early interventions for SAD, but also other mental disorders by targeting transdiagnostic constructs.

## Additional file

**Additional file 1.** A: Management of missing data. B: Preliminary analysis on minimization/denial. C: Intercorrelations across demographic characteristics and childhood adversities. D: Frequencies and severities of child maltreatment assessed with the CTQ in other studies. E: Means and standard deviations of childhood adversity severities across groups

## Data Availability

The datasets generated during and/or analysed during the current study are not publicly available to protect the anonymity of participants. Excerpts of the data are available from the corresponding author on reasonable request.

## Abbreviations

AD-: Anxiety disorder only
CTQ: Childhood Trauma Questionnaire
DEP-: Depressive disorder only
DSM-IV TR: Diagnostic and Statistical Manual of Mental Disorders
FBS: Fragebogen zu belastenden Sozialerfahrungen in der Peer Group (English: The Questionnaire of Aversive Social Experiences in the Peer Group)
GAD: Generalized anxiety disorder
GAD-: Generalized anxiety disorder only
OD+/−: Other mental disorder with or without comorbidity
SAD-: SAD only
SAD: Social anxiety disorder
SAD+/−: Social anxiety disorder with our without comorbidity
SAD+DEP: SAD with a comorbid depressive disorder
SCID I: Structured Clinical Interview for DSM-IV
SIAS: Social Interaction Anxiety Scale
SP: Specific phobia
SP-: Specific phobia only
SPAI: Social Phobia and Anxiety Inventory
SPS: Social Phobia Scale
