# Supplementary material for: Child maltreatment, peer victimization, and social anxiety in adulthood: a cross-sectional study in a treatment-seeking sample
Source: BMC Psychiatry. 2019 Dec 27;19:418. doi: 10.1186/s12888-019-2400-4 (PMC6935074; doi:10.1186/s12888-019-2400-4)
Supplement: Supplementary file 1 — Additional file 1. A: Management of missing data. B: Preliminary analysis on minimization/denial. C: Intercorrelations across demographic characteristics and childhood adversities. D: Frequencies and severities of child maltreatment assessed with the CTQ in other studies. E: Means and standard deviations of childhood adversity severities across groups [file 12888_2019_2400_MOESM1_ESM.docx]

**Child Maltreatment, Peer Victimization, and Social Anxiety in Adulthood:**

**A cross-sectional study in a treatment-seeking sample**

Antonia Brühl^1,2^, Hanna Kley^3^, Anja Grocholewski^2^, Frank Neuner^3^ & Nina Heinrichs^1,2^

^1^University of Bremen

^2^University of Braunschweig, outpatient clinic

^3^Bielefeld University, outpatient clinic

**Additional file 1**

Additional file 1A: Management of missing data

Additional file 1B: Preliminary analysis on minimization/denial

Additional file 1C: Intercorrelations across demographic characteristics and childhood adversities

Additional file 1D: Frequencies and severities of child maltreatment assessed with the CTQ in other studies

Additional file 1E: Means and standard deviations of childhood adversity severities across groups

1. **Management of missing data**

The available sample sizes of patient groups (i.e. SAD+/-, OD+/-, SAD-, GAD-, SP-, AD-, DEP-, AD+DEP) varied across outcome variables, i.e. emotional abuse, physical abuse, sexual abuse, physical neglect, emotional neglect, and peer victimization, due to missing data. Missing data resulted, for example, from patients’ refusals to complete all questionnaires or skipping some items on one or more scales. There were 2.7 % missing for emotional abuse, 1.1 % for physical abuse, 1.7 % for sexual abuse, 3.0 % for emotional neglect 1.7 % for physical neglect, and 5.9 % for peer victimization. A significant Little’s MCAR test, *χ^2^*(85) = 151.06, *p* < .001, revealed that the data were not missing completely at random. In order to investigate whether patients with missing values on one scale have higher severities of childhood adversities on other scales, group differences between patients with and without missing values were tested for each outcome.

Results of independent t-tests showed that patients with missing values for emotional abuse showed significant higher severities of emotional neglect (t = -3,16, p =.002) and physical neglect (t = -2.61, p = .015) compared to patients without missing values. Patients with missing values for physical abuse reported significant more emotional abuse (t = -3.46, p = .001). Patients with missing values for sexual abuse showed higher severities of emotional abuse (t = -5.03; p <.001), physical abuse (t = -2.31, p=.036), physical neglect (t= -3.54, p = .003), and peer victimization (t= -4.14, p <.001). Patients with missing values for physical neglect reported significant less physical abuse (t = 3.07, p = .006) compared to patients without missing values. Patients with and without missing values for emotional neglect or peer victimization did not differ in their severities of adversities across all types.

Findings indicate that missing values are not missing completely at random, but that patients with missing values on the emotional abuse, physical abuse, and sexual abuse scales reported significant higher maltreatment severities on other maltreatment scales. Therefore, we assume that some patients with missing data may actually be patients, who experienced severe maltreatment, but refused to answer single items, due to factors such as social desirability, shame, or embarrassment. Due to this systematically missing of data, we did not impute missing data in our study.

1. **Preliminary analysis on minimization/denial**

Although the CTQ is one of the most widely-used self-report measure of child maltreatment, scores on its minimization/denial (MD) subscale are usually omitted or not reported in the vast majority of previous studies (MacDonald et al., 2016). The MD subscale consist of 3 items indicating a positive response bias. For the MD items, scores of 1 through 4 were coded as 0 and scores of 5 were coded as 1. A score greater than 0 indicated minimization/denial.

MD is common across samples (about 10% - 40%) and scores may identify certain patient’s CTQ results for further investigation, analyses, or exclusion (MacDonald et al., 2015; MacDonald et al., 2016). In our study, 22% of the total sample endorsed one or more MD items, indicating that the reported maltreatment rates may actually underestimate the true rates. However, the rate we found in our study is lower compared to most rates in other samples (MacDonald et al., 2016). For example, in a 24-multinational sample , MacDonald and colleagues found that MD occurred in 28% of the CTQ scales from clinical subjects and 42% of scales from community subjects. Therefore, MD is a widespread phenomenon across clinical and non-clinical samples, so that we did not excluded these patients indicating a response bias. Instead, we compared MD mean scores across the patient groups used for our main analyses and found that MD severity did not significantly differ across patient (see Table B1), indicating that the potential respondent biases did not affect our main findings.

Table B1

*Group differences in CTQ minimization/denial across patient groups*

| Variable | **SAD-** | |  | **GAD-** | |  | **SP-** | |  | $\mathbf{Kruskal Wallis Test}$ | | |
| --- | --- | --- | --- | --- | --- | --- | --- | --- | --- | --- | --- | --- |
| Minimization/  denial | *M* | *(SD)* |  | *M* | *(SD)* |  | *M* | *(SD)* |  | *H* | *df* | *p* |
|  | 0.29 | (0.59) |  | 0.60 | (0.91) |  | 0.46 | (0.78) |  | 1.09 | 2 | .581 |
|  | **AD-** | |  | **DEP-** | |  |  | |  | **T-Test** | | |
| Minimization/  denial | *M* | *(SD)* |  | *M* | *(SD)* |  |  |  |  | *T* | *df* | *p* |
|  | 0.44 | (0.76) |  | 0.32 | (0.65) |  |  |  |  | 1.11 | 206 | .268 |
|  | **SAD-** | |  | **SAD+DEP** | |  |  | |  |  |  |  |
| Minimization/  denial | *M* | *(SD)* |  | *M* | *(SD)* |  |  |  |  | *T* | *df* | *p* |
|  | 0.29 | (0.59) |  | 0.56 | (0.59) |  |  |  |  | .25 | 109 | .802 |
|  | ***SAD+/-*** | |  | ***OD+/-*** | |  |  | |  |  |  |  |
| Minimization/  denial | M | (SD) |  | M | (SD) |  |  |  |  | *T* | *df* | *p* |
|  | 0.27 | (0.59) |  | 0.30 | (0.63) |  |  |  |  | -.47 | 791 | .637 |

*Note. M = Mean, SD = Standard Deviation,* SAD- = Social Anxiety Disorder only, GAD- = Generalized Anxiety Disorder only, SP- = Specific Phobia only, AD- = Anxiety Disorders only, DEP-= Depressive Disorders only, SAD+DEP = with SAD and a comorbid depressive disorder, SAD+/- = SAD with or without any comorbidity, OD+/- = any other mental disorder with or without comorbidity. Minimization/denial assessed with the CTQ.

**References:**

MacDonald, K., Thomas, M. L., MacDonald, T. M., & Sciolla, A. F. (2015). A perfect childhood? Clinical correlates of minimization and denial on the Childhood Trauma Questionnaire. *Journal of interpersonal violence*, *30*(6), 988-1009.

MacDonald, K., Thomas, M. L., Sciolla, A. F., Schneider, B., Pappas, K., Bleijenberg, G., ... & Dannlowski, U. (2016). Minimization of childhood maltreatment is common and consequential: results from a large, multinational sample using the childhood trauma questionnaire. *PLoS One*, *11*(1), e0146058.

1. **Intercorrelations across demographic characteristics and childhood adversities**

Bivariate analyses showed positive intercorrelations among types of child maltreatment as well as associations between all forms of child maltreatment and peer victimization, ranging from r = .22, *p* < .001 (emotional neglect) to .49, *p* < .001 (emotional abuse) in the total sample. While all forms of child maltreatment were positively associated with age, a negative association appeared for peer victimization and age (r = -.14, *p* < .001, see Table C1).

Table C1

*Spearman´s Intercorrelations across Demographic Characteristics, forms of Child Maltreatment, and Peer Victimization*

|  | | | PA | SA | EN | PN | PV | Age | Sex |
| --- | --- | --- | --- | --- | --- | --- | --- | --- | --- |
|  | Emotional abuse |  | .57** | .38** | .48** | .46** | .49** | .03 | .16** |
|  | Physical abuse |  |  | .39** | .37** | .40** | .34** | .17** | -.00 |
|  | Sexual abuse |  |  |  | .24** | .29** | .24** | .09** | .21** |
|  | Emotional neglect |  |  |  |  | .66** | .22** | .15** | .05 |
|  | Physical neglect |  |  |  |  |  | .25** | .17** | .04 |
|  | Peer victimization |  |  |  |  |  |  | -.14** | -.01 |
|  | Age |  |  |  |  |  |  |  | -.06 |

*Note.* *. Correlation is significant at the 0.05 level (2-tailed). ** Correlation is significant at the 0.01 level (2-tailed). PA = Physical abuse, SA = Sexual abuse, EN = Emotional neglect, PN = Physical neglect, PV = Peer victimization. *N*’s range from 1075 to 1085 due to occasional missing data. For sex: 0 = male, 1 = female

1. **Frequencies and severities of child maltreatment assessed with the CTQ in other studies**

Table D1

*Frequencies and severities of Child maltreatment assessed with the CTQ in Social Anxiety Disorder in the Present Study, previous Clinical Studies and a representative Sample of the German Population*

|  |  | Emotional Abuse | |  | Physical Abuse | |  | Sexual Abuse | |  | Emotional Neglect | |
| --- | --- | --- | --- | --- | --- | --- | --- | --- | --- | --- | --- | --- |
| Study | *N* | *M (SD)* | *%* |  | *M (SD)* | *%* |  | *M (SD)* | *%* |  | *M (SD)* | *%* |
| Iffland et al., 2013  - representative population sample | 2500 | 6.5 (2.6) | 10.2 |  | 5.9 (2.2) | 12.0 |  | 5.5 (1.7) | 6.2 |  | 10.1 (4.2) | 13.9 |
| Wingenfeld et al., 2010  - Anxiety | 69 | 8.9 (4.2) | N.A. |  | 6.2 (2.4) | N.A. |  | 5.6 (2.1) | N.A. |  | 11.9 (5.2) | N.A. |
| - Depression | 529 | 9.9 (5.4) | N.A. |  | 7.4 (4.1) | N.A. |  | 6.3 (3.8) | N.A. |  | 13.0 (5.9) | N.A. |
| Bruce et al., 2012  - SAD+/- | 156 | 10.9 (5.2) | 50.6 |  | 7.5 (3.5) | 34.6 |  | 9.0 (3.5) | 68.4 |  | 12.7 (5.0) | 36.5 |
| Bruce et al., 2013  - SAD+/- | 68 | 10.3 (4.9) | 44.3 |  | 6.9 (3.4) | 17.1 |  | 5.6 (1.6) | 10.0 |  | 12.7 (5.1) | 37.1 |
| Kuo et al., 2011.  - SAD+/- | 102 | N.A. | 51.0 |  | N.A. | 30.4 |  | N.A. | 17.6 |  | N.A. | 36.3 |
| Michail & Birchwood, 2014  - SAD+/- | 51 | N.A. | 45.0 |  | N.A. | 11.7 |  | N.A. | 17.7 |  | N.A. | 41,2 |
| Simon et al., 2009  - SAD+/- | 103 | N.A. | 56.3 |  | N.A. | 28.2 |  | N.A. | 17.5 |  | N.A | 38.9 |

*Note*. *M* = Mean, *SD* = Standard Deviation, *%* = Percentage of participants meeting the threshold for clinical significance, CTQ = childhood trauma questionnaire. Threshold for clinical significance in CTQ established by Walker et al. [40]. SAD- = Social anxiety disorder only, SAD+/- = Social anxiety disorder with or without comorbidity. N.A. = information was not reporte

1. **Means and standard deviations of childhood adversity severities across groups**

Table E1

*Means and standard deviations of childhood adversity severities across patients with social anxiety disorder only, generalized anxiety disorder only, and specific phobia only*

|  | SAD- | | |  | GAD- | | |  | SP- | | |
| --- | --- | --- | --- | --- | --- | --- | --- | --- | --- | --- | --- |
| *Childhood adversities* | *n* | *M* | *(SD)* |  | *n* | *M* | *(SD)* |  | *n* | *M* | *(SD)* |
| Emotional abuse | 25 | 8.12 | (2.88) |  | 19 | 8.11 | (4.19) |  | 18 | 7.83 | (3.19) |
| Physical abuse | 25 | 5.24 | (0.72) |  | 19 | 5.53 | (2.06) |  | 18 | 6.00 | (2.45) |
| Sexual abuse | 25 | 5.04 | (0.20) |  | 19 | 5.16 | (0.69) |  | 18 | 5.17 | (0.51) |
| Emotional neglect | 25 | 11.14 | (3.95) |  | 19 | 11.79 | (6.70) |  | 18 | 11.78 | (4.51) |
| Physical neglect | 25 | 6.18 | (1.74) |  | 18 | 6.22 | (1.70) |  | 17 | 7.06 | (2.33) |
| Peer victimization | 24 | 6.54 | (5.21) |  | 18 | 10.22 | (8.98) |  | 18 | 8.61 | (5.37) |

*Note.* SAD- = Social Anxiety Disorder only, GAD- = Generalized Anxiety Disorder only, SP- = Specific Phobia only, *M =* Mean*, SD* = Standard Deviation*.* Emotional abuse, physical abuse, sexual abuse, emotional neglect, and physical neglect assessed with the CTQ. Peer victimization assessed with the FBS.

Table E2

*Means and standard deviations of childhood adversity severities in patients with anxiety disorders only and depressive disorders only*

|  | AD- | | |  | DEP- | | |
| --- | --- | --- | --- | --- | --- | --- | --- |
| *Childhood adversities* | *n* | *M* | *(SD)* |  | *n* | *M* | *(SD)* |
| Emotional abuse | 62 | 8.03 | (3.36) |  | 236 | 9.97 | (4.91) |
| Physical abuse | 62 | 5.54 | (1.80) |  | 238 | 6.40 | (2.84) |
| Sexual abuse | 62 | 5.11 | (0.48) |  | 238 | 5.90 | (2.52) |
| Emotional neglect | 62 | 11.52 | (5.01) |  | 237 | 13.06 | (5.26) |
| Physical neglect | 62 | 6.44 | (1.92) |  | 239 | 8.31 | (3.36) |
| Peer victimization | 60 | 8.27 | (6.67) |  | 227 | 11.30 | (7.58) |

*Note.* AD- = Social Anxiety Disorder only, Generalized Anxiety Disorder only, Specific Phobia only; DEP- = depressive episode only, recurrent depressive disorder only, or dysthymia only); *M =* Mean*, SD* = Standard Deviation*.* Emotional abuse, physical abuse, sexual abuse, emotional neglect, and physical neglect assessed with the CTQ. Peer victimization assessed with the FBS.

Table E3

*Means and standard deviations of childhood adversity severities in patients with social anxiety disorder only and social anxiety disorder with a comorbid depressive disorder*

|  | SAD- | | |  | SAD+DEP | | |
| --- | --- | --- | --- | --- | --- | --- | --- |
| *Childhood adversities* | *n* | *M* | *(SD)* |  | *n* | *M* | *(SD)* |
| Emotional abuse | 25 | 8.12 | (2.88) |  | 138 | 11.63 | (5.74) |
| Physical abuse | 25 | 5.24 | (0.72) |  | 143 | 7.44 | (4.03) |
| Sexual abuse | 25 | 5.04 | (0.20) |  | 143 | 6.92 | (4.64) |
| Emotional neglect | 25 | 11.14 | (3.95) |  | 138 | 14.17 | (5.95) |
| Physical neglect | 25 | 6.18 | (1.74) |  | 142 | 8.72 | (3.36) |
| Peer victimization | 24 | 6.54 | (5.21) |  | 136 | 15.14 | (8.45) |

*Note. S*AD- = Social Anxiety Disorder only, SAD+DEP- = Social Anxiety Disorder with a comorbid depressive disorder; *M =* Mean*, SD* = Standard Deviation*.* Emotional abuse, physical abuse, sexual abuse, emotional neglect, and physical neglect assessed with the CTQ. Peer victimization assessed with the FBS.
